# Supplementary figures and images for: Machine learning detects hidden treatment response patterns only in the presence of comprehensive clinical phenotyping
Source: PLoS One. 2025 Oct 21;20(10):e0334858. doi: 10.1371/journal.pone.0334858 (PMC12539744; doi:10.1371/journal.pone.0334858)

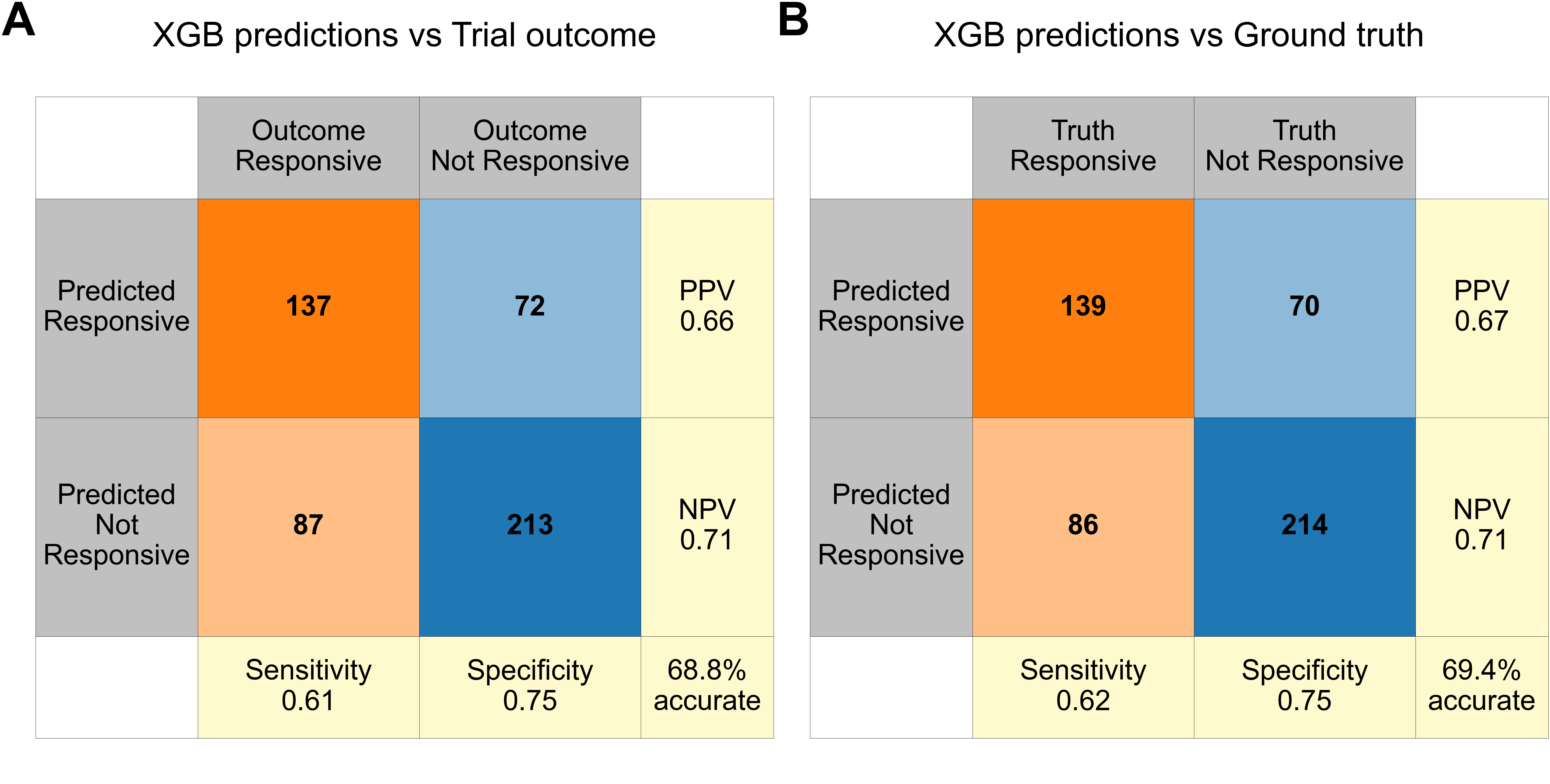


**S1 Fig**

Supplement: S1 Fig — Predictions of treatment response using XGB analysis are compared with the treatment response apparent in the trial outcome measure (A) and the ground truth (B). Orange shading denotes treatment responsive (suggested in the trial outcome or from ground truth) and blue shading denotes non-treatment responsive cells; bold orange/blue denote correct treatment allocation according to the outcome; light orange/blue denote inappropriate treatment allocation. PPV: positive predictive value; NPV: negative predictive value. (DOCX) [file pone.0334858.s001.docx]
