# Supplementary material for: Machine learning detects hidden treatment response patterns only in the presence of comprehensive clinical phenotyping
Source: PLoS One. 2025 Oct 21;20(10):e0334858. doi: 10.1371/journal.pone.0334858 (PMC12539744; doi:10.1371/journal.pone.0334858)
Supplement: Attachment — Submitted filename: Response to reviewers.docx [file pone.0334858.s002.docx]

We thank the reviewer for their helpful comments and appreciate the chance to refine our manuscript. We have carefully considered each suggestion and below are our point-by-point responses, detailing the revisions incorporated into the manuscript based on the valuable feedback.

***Reviewer #1: In this work, a randomised clinical trial has been stimulated to compare gradient boosting (XG boost) machine learning with traditional analysis when “ground truth” treatment responsiveness depends on the interaction of multiple phenotypic variables. The detected results from traditional analysis indicated that outcome measure change from baseline was 4.23 (95% CI 3.64–4.82) of patients. In contrast, the treatment response of patients reached at 97.8% (95% CI 96.6–99.1) in the context of machine learning. Notably, analysis results drop to 69.4% (95% CI 65.3–73.4) because of an omitted single variable. These indicated that machine learning could be maximized insights derived from clinical research studies. Overall, it is meaningful work, but several points should be clarified.***

***1. The introduction did not sufficiently highlight the innovation and superiority of this work. The authors are encouraged to clarify the innovation and superiority of their work.***

We have now revised the Introduction (final paragraph) to explicitly highlight the core, novel aspect of the current work, as follows:

*“In this study, we investigated how both the analysis approach and characterisation of clinical phenotype within an RCT influences our ability to infer underlying 'ground truth'. Using simulated data representative of typical RCTs, we conducted a novel, formal comparison contrasting the effectiveness of traditional statistical methods versus ML approaches at identifying the key factors and interactions driving treatment response.”*

When coupled with the added context regarding existing clinical ML applications (addressing comment 7, below), we believe the manuscript now articulates its specific place and contribution within the field more clearly.

**2. Clarify methodological generalizability and limitations of ml approaches. While the discussion provides a strong case for the superiority of XGB in this particular context, the manuscript would benefit from a more balanced and critical evaluation of the generalizability of these findings. For example, the authors could elaborate on the types of datasets or clinical conditions where ML approaches like XGB may not outperform traditional methods, as well as practical constraints such as data availability, model interpretability, or computational requirements. This would strengthen the manuscript by providing a more nuanced view of ML’s utility across diverse clinical settings.**

We agree with the need for balanced discussion regarding the generalisability and limitations of ML approaches beyond these specific findings with XGB. In addition to the existing acknowledgement of the distinct strengths of traditional inferential statistics (paragraph 3, sentence 4), we have now explicitly incorporated the recommendation to elaborate on the types of datasets of clinical conditions where ML approaches may not outperform traditional methods (same laster in the same third paragraph):

*“In many circumstances, ML approaches may not outperform traditional methods, especially if treatment response is related to simple, univariate and/or linear trends; if datasets are very small where complex models risk overfitting; or when strong theoretical knowledge already provides a robust explanatory framework.”*

Regarding the importance of considering practical constraints, the Discussion addresses multiple key components of these practical considerations: challenges related to data availability and collection (Discussion paragraph 8), considerations surrounding model interpretability (Results section 4 and Discussion paragraph 11), and computational requirements (Discussion paragraph 13).

Together, these sections provide a balanced and comprehensive summary of some key considerations and limitations with ML relative to traditional inferential approaches.

***3. Elaborate on the nature and impact of clinical phenotyping. The manuscript emphasizes the importance of “comprehensive clinical phenotyping,” but does not provide sufficient detail about what this entails in practice. The authors are encouraged to elaborate on the specific phenotypic variables critical to the model’s success and discuss how such detailed phenotyping can be realistically obtained in real-world clinical trials. Providing concrete examples or frameworks would enhance the translational relevance and practical guidance for future research.***

We agree with the reviewer on the importance of clearly outlining what is meant by 'comprehensive clinical phenotyping’ in practice. To ensure this concept is clear from the outset, the second and third sentences of the Introduction orient the reader with an explanation of our use of the term 'clinical phenotype' and how these might be practically expanded to be more comprehensive .

Regarding the specific phenotypic variables critical to the model’s success in this study, there are two aspects to this: the predefined 'ground truth' variables and those identified empirically by the analysis. We hope the following locations clearly provide this detail: The 'ground truth' variables underpinning our simulations are presented visually in Figure 1 and described textually in the first sections of the Results and Methods. Subsequently, the variables identified as most important by the XGBoost analysis are presented and discussed in the Results section associated with Figures 4 and 5. Please let us know if there is any specific aspect which requires further explanation.

We acknowledge the practical challenges of acquiring detailed phenotypic data in real-world clinical trials. The specifics are highly dependent on the context of each study, making comprehensive coverage in a general discussion difficult. We address these practical considerations across several paragraphs in the Discussion (specifically 4, 5, 6, and 8), covering aspects such as utilising existing clinical records and the potential need for enhanced data capture structures. To provide a concrete example, as requested, paragraph 5 includes a description using stroke trials as an example comparing a relatively reductive NIHSS score with nuanced information which might be available within a clinical history (e.g., that which might indicate the presence of potential cerebral amyloid angiopathy) and impact treatment outcomes. Prompted by the reviewer's feedback, we have added clearer emphasis in paragraph 5:

*“The precise phenotypic variables which might be critical will vary dependent upon the specific circumstances of an investigation, but for one specific example, highly impactful and rigorous trials in stroke medicine often reduce a complex clinical syndrome to a quantified NIH Stroke Scale (NIHSS) score, time since symptom onset, and a limited number of comorbidities such as diabetes, atrial fibrillation, and hypertension. However, critical nuances in the clinical history, such as previous recurrent episodes which might indicate cerebral amyloid angiopathy, which can increase the risk of haemorrhagic complications from thrombolysis, may be overlooked in initial trials and can take several years to become apparent.”*

***4. The simulation assumes linear effects for non-critical variables (V1/V2), which may oversimplify real-world clinical complexity. For instance, age or biomarkers rarely exhibit uniform noise distributions. Introducing realistic correlations or nonlinear interactions among variables would enhance ecological validity. Additionally, XGBoost hyperparameters (e.g., max depth=3) are justified but lack sensitivity analysis. Reporting performance variation with deeper trees or alternative tuning strategies (e.g., grid search) would strengthen confidence in model robustness. Subgroup analyses in Figure 2 lack multiplicity adjustments, inflating Type I error risks. Acknowledging this limitation and proposing Bonferroni corrections or false discovery rate control would improve rigor.***

Regarding the reviewer's concerns about the potential oversimplification of assuming purely linear effects for the V1/V2 non-critical variables, we acknowledge that real-world clinical variables often exhibit complex, non-linear relationships and non-uniform noise distributions. While V1/V2 were kept simple for illustrative clarity, we designed the broader simulation involving up to 10,000 further non-critical variables specifically to address this concern by incorporating a variety of covariance structures, including both linear and non-linear interactions. We believe this approach significantly enhances the ecological validity beyond the baseline V1/V2 example. We recognise, as the reviewer implies, that comprehensively modelling all potential real-world biological complexity is an immense challenge, likely varying greatly between specific clinical scenarios. We believe the current simulation strikes a reasonable balance, providing a robust demonstration of the core concepts while acknowledging that specific applications would necessitate tailoring the modelled interactions to different specific contexts.

Our intention with the current manuscript was primarily to provide a proof-of-concept introduction to these ML methods for a predominantly clinical audience, hence our initial use of common default hyperparameters (like max depth = 3) to maintain focus on the core message. However, we are in full agreement that rigorous hyperparameter optimisation is a crucial step in developing deployable ML models. Therefore, following the reviewer's suggestion, we have now added text explaining this point to the Discussion paragraph 13:

*“Applying these techniques effectively in practice requires systematic hyperparameter tuning (e.g., grid search, random search, Bayesian optimisation) and sensitivity analysis to refine model performance. Although a detailed exploration is outside this work's scope, practical guidance on these techniques is readily available in other resources.”*

The new text is referenced to guide interested readers towards practical resources for exploring these optimisation strategies in greater depth.

Regarding multiplicity adjustments, we agree with the reviewer’s point, and this motivated our choice to present results in Figure 2 with only 95% confidence intervals rather than using significance of non-corrected statistical tests. We have fully incorporated the reviewer’s recommendation to propose Bonferroni corrections or false discovery rater control in the Discussion (paragraph 6):

*Using univariate statistical analysis, multiple comparisons across subgroups increases the risk of identifying spurious associations, and necessitates adjustments to statistical significance thresholds such as Bonferroni correction or false discovery rater control, which can obscure genuine trends.*

It is interesting to note that even with the potential inflated type I error risk, traditional methods still did not detect effects which are clear with ML analysis.

***5. The term "hidden treatment patterns" risks overstating novelty, as interactions are often hypothesized in clinical research. Clarify whether the "ground truth" represents simulated relationships or reflects established biological mechanisms (e.g., pharmacogenomics). Confusion matrices (Figure 3) use a colorblind-unfriendly red/green palette, risking misinterpretation. Replacing these with patterned fills or dual-labeling (e.g., text annotations) would improve accessibility. Reporting additional metrics like Cohen’s kappa or area under the receiver operating characteristic curve (AUROC) would contextualize accuracy improvements over chance expectations.***

Regarding use of the term ‘hidden treatment patterns’: We agree that interactions are often hypothesised in clinical research. However, our intention with this work is to consider the specific scenario where complex relationships that might *not* form part of *a priori* hypotheses might be uncovered through use of data-driven methods applied post hoc, as discussed in paragraph 12 of the Discussion.

Regarding clarification of what the ‘ground truth’ represents; we confirm this was indeed simulated to create a controlled environment for evaluating the methods. We have now added explicit statements clarifying this in both the Results (first section, paragraph 4):

*“A simulated non-linear relationship between these three variables (X, Y, Z) determines which patients will be responsive to the treatment”*

and Methods (section 1, paragraph 4):

*“Whether or not a patient would be responsive to the treatment was determined by a simulated non-linear multivariable relationship”*

Regarding metric reporting, we carefully considered the most appropriate metrics for illustrating the key points within the intended clinical context and opted to use confusion matrices. This choice was motivated by several factors: confusion matrices directly reflect performance at specific decision thresholds pertinent to clinical practice and the binary treatment responsive/non-responsive dichotomy here; they provide a highly intuitive breakdown of absolute true positive, false positive, false negative, and true counts crucial for interpreting clinical impact; and they allow clear evaluation of performance on potentially small but significant minority classes often found in imbalanced clinical datasets (e.g. those discussed in Results, section 3, paragraph 1, final sentence). None of these key comparisons would be possible with AUROC, especially for this work’s binary prediction. Cohen’s Kappa, while excellent for assessing inter-rater reliability, is not applicable in this specific work which compares analysis outputs with a defined ground truth.

We are grateful for the reviewer highlighting the significant accessibility issue with the original red/green colour scheme in Figure 3. We have completely revised the colour scheme for not just Figure 3, but also Figure 1, Figure 5 and Supplementary Figure 1. The problematic red/green combination has been replaced with a widely recommended colourblind-friendly blue/orange scheme. To maintain clear visual distinction, in Figure 4, the shades of blue previously used have been changed to shades of red.

***6. The study fails to connect findings to real-world clinical scenarios where phenotypic complexity limits trial generalizability (e.g., oncology, neurology). For example, how might missing variables like comorbidities or genomic markers affect ML predictions in diverse populations? Overstating ML’s ability to "maximize insights" without discussing computational costs, reproducibility challenges, or ethical implications (e.g., algorithmic bias in treatment allocation) undermines clinical utility. Underexplored potential biases (e.g., over-reliance on correlated variables like V1/V2) and the absence of temporal dynamics (longitudinal outcomes) limit translational relevance.***

Regarding the connection to real-world clinical scenarios, phenotypic complexity, and the impact of missing variables, we address this directly in the third paragraph of the Introduction. We provide referenced, established concrete examples from clinical practice where initial treatment strategies were suboptimal precisely because key phenotypic variables within diverse populations were initially 'missing variables' from analyses, taking decades to refine our understanding. These specific real-world examples include the use of aspirin in cardiovascular disease, beta-blockers in heart failure, calcium channel blockers in hypertension, and antidepressants in mood disorders. One of the key analyses is to demonstrate the impact of even a single missing variable (in this instance ‘Z’ which is analogous to any potential comorbidity of genomic marker in a diverse population) upon ML predictions (Results section 5).

We agree fully with the reviewer that it is crucial not to overstate the capabilities of ML and to acknowledge its limitations. Concerning computational costs, we include a discussion of the resources used for all analyses presented in this work within the Discussion section (paragraph 8). The core premise of our study inherently addresses reproducibility - comparing ML outputs directly against a known simulated 'ground truth' i.e. whether methods can recover and reproduce known patterns under controlled conditions. We further support reproducibility of our results by providing full open access to the code and data described throughout the manuscript.

Regarding consideration of potential biases, we include formal consideration of biases related to the use of standard versus ML analysis at multiple points throughout the manuscript (Results, section 3, paragraph 1; Discussion paragraph 2; Discussion paragraph 15) and how specific patient characteristics and subgroups might be systematically disadvantaged by the way in which data are analysed.

Regarding longitudinal temporal dynamics, this work considers the standard practice in RCTs of assessing outcomes at a defined primary endpoint. Consideration of longitudinal temporal dynamics falls outside the scope of this targeted simulation of common methodology typical used in cross-sectional clinical trials.

***7. Claiming "data excess has no penalty" risks overgeneralization, as sparser signals or smaller datasets may suffer from noise-induced overfitting. Temper conclusions with caveats about scalability. The manuscript lacks engagement with existing literature on ML in clinical trials (e.g., SHAP/XGBoost applications in healthcare). A brief review contextualizing contributions (e.g., comparing to rule-based decision trees) would strengthen its novelty.***

Regarding the reviewer's concern about the phrase "data excess has no penalty," we wish to clarify that this specific statement (or a similar statement) does not appear in our manuscript. We understand the reviewer's underlying point though, and have been careful to avoid these sorts of overgeneralisation, choosing instead to make factual statements describing the results of the analysis.

When mentioning "scalability," we are referencing a key algorithmic feature of XGBoost, as highlighted in its original description by Chen & Guestrin (reference 18 in the bibliography), related to its computational efficiency with large datasets.

We also thank the reviewer for the recommendation to strengthen the connection to the existing literature on machine learning applications in clinical trials. As such, we have now included reference to comprehensive review articles within the Introduction (paragraph 4).

*Indeed, a growing amount of clinical research includes the use of ML techniques and considers their current limitations when applied in clinical settings [14,15]. The effectiveness of ML models, as for all methodologies, critically depends on the quality and quantity of the data available to them [16].*

Furthermore, we have added specific citations regarding the application of XGBoost (Introduction, paragraph 5).

*We then examined the capability of ML to uncover additional insights from the same data. We used XGBoost (XGB), a form of gradient boosting ML which has demonstrated state-of-the-art performance on a range of problems involving complex, non-linear interactions between variables^17,18^, and has been applied in a variety of clinical settings^19–22^.*
