# Supplementary material for: Machine learning detects hidden treatment response patterns only in the presence of comprehensive clinical phenotyping
Source: PLoS One. 2025 Oct 21;20(10):e0334858. doi: 10.1371/journal.pone.0334858 (PMC12539744; doi:10.1371/journal.pone.0334858)
Supplement: Attachment — Submitted filename: Response to reviewer comments.docx [file pone.0334858.s003.docx]

**Reviewer #1: The authors have meticulously addressed all the concerns raised in the initial review. I recommend that this manuscript be accepted for publication in PLOS One.**

*We thank the reviewer again for their previous comments. We are pleased they are satisfied with the amendments.*

**Reviewer #2: The authors took the reviewers' comments into account.**

**However, the database simulation seems unrealistic.
First, it is impossible to have a database based only on gender and age as sociodemographic factors and on only five variables.
Furthermore, the age variable generally has a normal distribution. It is illogical to treat it as a uniform distribution and assign the same probability to a 100-year-old patient as to an 18-year-old patient.
How did the authors also choose the lower and upper bounds for each simulated variable?**

**We are in the era of open science, and there are certainly several databases in the context studied, drawn from the real world. Why didn't the authors consider concretizing their work by testing it on a real database (such as the dbGaP example)?**

*We thank the reviewer for their comments.*

*Regarding choice of distributions and bounds, the variables were designed to be arbitrary placeholders to demonstrate a principle. We used uniform distributions for their simplicity and to make the fewest possible assumptions as a basis for generating random numbers. The variable labels "age" and "sex" were chosen purely to anchor the reader with familiar concepts for continuous and categorical data, respectively. We are not making any claims about clinical effects in specific age groups or modelling real-world demographics. Our choice of a uniform distribution for the variable labelled "age" was to demonstrate an effect of randomly generated numbers within a continuous variable, not to model the distribution of human age.*

*To ensure this is more clear to the reader, we have revised the manuscript. We have added the following lines to the Methods section [lines 582 - 585]:*

*“…we generated a set of arbitrary clinical phenotype variables. To minimise assumptions about the data's underlying distributions, each variable was modelled independently and drawn from uniform distributions.”*

*We have also clarified the arbitrary nature of the variable bounds by adding this sentence [line 594 - 595]:*

*“The lower and upper bounds for each variable were selected arbitrarily.”*

*Finally, we reinforce this point in the Results section with the following new text [lines 111 - 112]:*

*"For each patient, the clinical phenotype consists of multiple arbitrary clinical variables, including illustrative variables for..."*

*Regarding the number of variables, we intentionally restricted the number of variables to the minimum required to illustrate our core points. In drafting this manuscript, we found that models with more variables made the specific effects we were trying to highlight difficult for the reader to follow, without adding any new conceptual insights. The purpose was clarity of demonstration. To show that these principles are not limited to overly simplified scenarios, we included the analysis in Figure 6, which demonstrates that the same principles hold true in a model with much greater complexity, including a larger number of variables and covariance structures.*

*Regarding the use of a real-world database, the core purpose of this study was to demonstrate that certain machine learning techniques can detect treatment effects (and non-effects) that might be missed by traditional inferential statistics, we therefore chose simulated data to be able to program specific, known effects into the data and then definitively assess whether a given analytical technique can or cannot detect them. This provides a clear, unambiguous benchmark of the method's capabilities, which is not possible with a real-world dataset. Our aim here is to establish a principle of analytical sensitivity, which can then be applied to real-world data in future work.*

*We believe these revisions make the illustrative, non-realistic nature of our simulation explicit, directly addressing the reviewer's concerns and clarifying the methodological rationale for our readers.*

**An important criterion is missing: model power**

*We thank the reviewer for raising the important point about model power. This is an important criterion for establishing the reliability of the method, and we have now performed a new dedicated analysis to address it.*

*The analysis presented in Figure 3 was intended as a deep dive into the model's classification performance on a single, representative instance of our simulation. It demonstrates how the model works by more accurately identifying the ground truth than the noisy trial outcome but it does not establish the reliability of this success.*

*To address this, we have now conducted a formal power analysis to quantify the probability that our XGB approach will successfully detect the true treatment effect under the simulated conditions.*

*We defined "successful detection" in a given simulation run as the model achieving an accuracy of >90% against the known ground truth labels. We then ran our entire simulation and analysis pipeline 500 times, recording a success or failure for each run based on this criterion.*

*The results of this analysis show that the model's power was 100%. Specifically, in 500 out of 500 independent simulations, the XGB model achieved >90% accuracy against the ground truth, demonstrating that our method is highly reliable and not the result of a single, fortuitous outcome.*

*We have added details of this power analysis to the manuscript. In the Methods Machine learning analysis section [lines 676 - 679]:*

*“To quantify the statistical power and robustness of our analytical approach, the entire data generation and XGB analysis pipeline was repeated for 500 independent runs. For each run, a "successful detection" was defined as the model achieving an accuracy greater than 90% when classifying against the known ground truth.”*

*In the Results, Analysis of the same randomised control trial data using machine learning section [lines 237 - 239]:*

*“A power analysis revealed that the method was highly robust and a success criterion of >90% accuracy against ground truth was met in 100% of 500 simulation runs, resulting in a statistical power of 100% to detect the simulated effect under these conditions.”*

**For the LR model, why was the choice of L2 penalized? Since this is a simulation, an LR elastic model should have been used to determine the exact values of L1 and L2 that are preferable according to the simulated cohort.**

*Our choice of a standard L2-penalised model was a deliberate methodological decision, designed to create a robust and interpretable baseline for comparison against our primary XGB ML technique.*

*Our primary goal for the LR analysis was to create a strong, traditional statistical competitor. To this end, we did more than just fit a simple model; we explicitly enhanced its capability by including interaction terms between all combinations of variables. By giving the LR model the capacity to see these interactions, we sought to ensure a fair comparison with XGB.*

*With all variables and their interactions included, the primary risk to the model is overfitting due to multicollinearity and large coefficient values. L2 regularisation is precisely the standard and appropriate tool for managing this risk. It penalises large coefficients to prevent any single feature from dominating, which is exactly what is needed here. Therefore, a standard L2 penalty is the most parsimonious and directly applicable choice. We used the default, well-vetted parameters from the scikit-learn library. This approach represents a standard, reproducible, and widely understood implementation of logistic regression. Introducing a complex hyperparameter tuning process for the baseline model (as required by Elastic Net) would add a layer of optimisation that could obscure the core comparison of this study, which is focused on the fundamental differences between the analytical approaches, not on fine-tuning (and potential overfitting) a specific baseline model.*

*Our implemented LR model, enhanced with interaction terms and appropriately regularised with a standard L2 penalty, represents a powerful and fair baseline. It was intentionally configured this way to robustly test the hypothesis rather than being optimised for a single data instance. We believe this choice is methodologically sound and best serves the illustrative purpose of our study.*

*We have included mention of LR elastic models alongside discussion about other forms of hyperparameter tuning in the Discussion section [lines 513 - 517]:*

*“Applying these techniques effectively in practice requires systematic hyperparameter tuning (e.g., grid search, random search, Bayesian optimisation, LR elastic models) and sensitivity analysis to refine model performance. Although a detailed exploration is outside this work's scope, practical guidance on these techniques is readily available in other resources …”*

**Several studies have validated the power of XGBoost to predict the phenomena studied on real databases. However, an interpretability problem remains compared to its recognized flexibility. This problem is also present in the simulation carried out in this article. What is the added value of this work?**

*The reviewer is correct that while the predictive power of models like XGB is well-established, their perceived lack of interpretability has been a major barrier to clinical adoption.* *Addressing this very challenge is one of the central contributions of our work, i.e., providing a demonstration in how to deliver the clinical need for transparent, interpretable insights, rather than solely pursing predictive performance.*

*While many studies stop after reporting high accuracy, our paper's main purpose is to demonstrate a practical workflow for how this "black box" can be “opened” and its findings made useful to a clinician. We illustrate this through our application of post-hoc interpretability techniques, which allow us to move beyond a simple prediction and understand the drivers of that prediction.*

*We show how to determine the key factors driving the model's predictions across the entire cohort. For example, our analysis of feature importance in Figure 4 identifies which clinical phenotypes the model relied on most heavily, enabling clinicians to see whether biologically plausible factors are driving results and providing potentially hypothesis generating novel insights.*

*The added value is not necessarily in proving, once again, that XGB is powerful. The added value is in providing an illustrative template in how it can complement and directly address the flaws in standard inferential techniques used for clinical research. We demonstrate a practical methodology to translate a powerful but opaque prediction into a transparent, patient-specific insight that could guide clinical decision-making and hypothesis generation. Our work is intended to serve as a guide for researchers and clinicians on how to navigate the interpretability challenge, and to benefit from the full potential of these advanced models in medicine.*

*Our work also provides a practical demonstration of the data requirements needed for these methods to be reliable. We illustrate that the model's insights are highly sensitive to the completeness of the input data. The absence of even a single key feature can drastically alter the model’s performance, and the resulting clinical insights. This finding elevates the importance of comprehensive and robust patient phenotyping from merely "good practice" to an essential prerequisite for the safe and effective deployment of these advanced models.*

**It is important to note that the figures are of low resolution. Please increase it.**

*Thank you for noting the resolution issue. We have addressed this by providing a new, high-resolution version of Figure 4. All other figures have now been rendered at the maximum resolution and dimensions permitted by the journal's submission guidelines.*

**Reviewer #3: The methodology section used in this paper is missing. A well-developed and structured section is mandatory.

From the introduction section, we move on to the results section; without the methodology section, it is impossible to complete the article.**

*We direct the reviewer to the Methods section starting from page 14. The manuscript is structured in keeping with the journal’s manuscript organisation guidelines (*[*https://journals.plos.org/plosone/s/submission-guidelines*](https://journals.plos.org/plosone/s/submission-guidelines)*) whereby the Materials and Methods, Results and Discussion sections can be presented in any order. In the first paragraph of the Results section, we direct the reader to the Methods section for full methodological details.*

**Result section:
Title (lines 106-108): Too long as a title**

*We have revised this title to be more concise while retaining its core message. It now reads:*

*“Creation of simulated clinical cohort data and determinants of ‘ground truth’ treatment response”*

F**ig 1. Investigation outline line133 :
for figure 1: what is this explanatory and interpretative paragraph? make the interpretations of your text then put figure 1 in parentheses at the end of the paragraph**

*We have ensured that the Figure 1 legend adheres strictly to the journal’s figure caption requirements (*[*https://journals.plos.org/plosone/s/figures#loc-captions*](https://journals.plos.org/plosone/s/figures#loc-captions)*), which mandate that captions must be self-sufficient and fully understandable without reference to the main text. To comply with the requirement to 'Describe each part of a multipart figure,' the current legend structure is necessary. Moving descriptive text elsewhere would compromise its standalone clarity and violate these guidelines.*

**title : An analysis of the randomised control trial data using traditional inferential statistics (lne 173 ) : Too long as a title**

*We have revised the section heading for brevity, ensuring it remains a clear guide to the content while maintaining consistency with other headings. It now reads:*

*“Analysis using traditional inferential statistics”*

*To maintain consistency, we also changed the title of the section which follows to:*

*“Analysis of the same data using machine learning”*

**Fig 2 (line 185-187) : is't title ?!**

**The same remarks for the rest of the figures and paragraphs**
*We have amended the caption for Figure 2 to clearly separate out a title and additional description in the legend. Upon reviewing all remaining figures, we confirmed that they were already consistent with this correct format, each featuring a distinct title. The issue was therefore seemingly isolated to Figure 2, which has now been rectified.*

*We were uncertain how the remark “is't (sic) title ?!” applies to 'paragraphs,' but if there is a specific formatting issue we have overlooked, we would be happy to address it.*

**Revise your text in the form of presenting the information so that it is easy for the reader to read**

*While we were encouraged that other reviewers complimented the writing, we recognise that clarity is paramount. In light of your feedback, we have carefully re-read the manuscript.* *It would be helpful to know if any lack of clarity stems from the writing itself, or perhaps from missing context which is detailed more fully in the Methods section which the reviewer’s first comment appears to indicate they had not been aware is included in the Manuscript. We would be grateful if you could point to any specific passages that are unclear, and we will happily revise them.*

**Reviewer #4: The revised manuscript "Machine learning detects hidden treatment response patterns only in the presence of comprehensive clinical phenotyping" is well written and an important contribution to the body of knowledge.**

**The authors responded well to all the criticisms and concerns raised by the reviewers and I feel the revised manuscript is fit for publication.**

*We thank the reviewer for their comments.*

**However, one minor observation is that the machine learning model as developed by the authors is generally more applicable to diseases without a definitive cause(s), for example degenerative conditions or chronic non-communicable diseases. I wonder how the model would be applied, for example, in case of an infection or infectious disease like malaria with a known cause that can be detected in a laboratory and used as a guide by clinicians in determining therapy? Because with most infections, once they are effectively treated, the symptoms like headache, vomiting, diarrhoea etc, disappear. In other words, can the authors provide a scope for the diseases where the model can best be applied?**

*We thank the reviewer for this important question about the model's scope. We agree that its application in chronic, non-communicable diseases is clear, and we appreciate the opportunity to elaborate on its utility for things like infectious diseases.*

*The 'treatment effect' our model analyses is not necessarily a binary outcome (e.g., recovery vs. death) but could just as easily be a continuous measure reflecting, for example, the rate of recovery. For an infectious disease, where the immune system would naturally work to resolve the infection, a model could assess treatment-related improvements in the speed of this recovery relative to a comparator group.*

*For example, in the case of malaria, the primary outcome measure might be 'time to symptom resolution' or 'avoidance of adverse outcomes.' Even with effective treatment, there can be significant treatment-related variance in these outcomes. This purpose of this kind of analytical approach could be to identify which patient phenotypes are associated with this heterogeneity, helping to predict which patients might have a slower or more complicated recovery.*

*We have added a new paragraph to the Discussion section to explain this important point [lines 547 - 555]:*

“*In clinical situations that appear to have a simple binary endpoint, such as survival or complete resolution of symptoms from an acute infection, a ML model's utility can come from considering more granular, secondary outcomes that capture the patient's recovery trajectory. For example, even when a treatment ensures patient survival, there is often significant heterogeneity in the time to symptomatic resolution, the incidence of treatment-related adverse events, or the duration of hospitalisation. By defining the 'treatment effect' in terms of these more nuanced metrics, ML analysis can still identify patient phenotypes associated with more or less favourable responses. The approach’s core strength lies in potentially helping explain any form of clinically relevant, treatment-related variance.*”
